# Differential response of oxidative and glycolytic skeletal muscle fibers to mesterolone

**DOI:** 10.1038/s41598-021-91854-4

**Published:** 2021-06-10

**Authors:** Hasan A. Asfour, Emad I. Shaqoura, Raed S. Said, Ayman G. Mustafa, Bright Starling Emerald, Mohammed Z. Allouh

**Affiliations:** 1 Department of Anatomy, Faculty of Medicine, Jordan University of Science and Technology, Irbid, Jordan; 2 Université Paris-Saclay, UVSQ, Inserm, END-ICAP, 78000 Versailles, France; 3 Basic Medical Science Department, College of Medicine, QU Health, Qatar University, Doha, Qatar; 4 Biomedical and Pharmaceutical Research Unit, QU Health, Qatar University, Doha, Qatar; 5 Department of Anatomy, College of Medicine and Health Sciences, United Arab Emirates University, Al Ain, UAE

**Keywords:** Animal physiology, Skeletal muscle, Muscle

## Abstract

Oxidative and glycolytic muscle fibers differ in their ultrastructure, metabolism, and responses to physiological stimuli and pathological insults. We examined whether these fibers respond differentially to exogenous anabolic androgenic steroids (AASs) by comparing morphological and histological changes between the oxidative *anterior latissimus dorsi* (ALD) and glycolytic *pectoralis major* (PM) fibers in adult avian muscles. Adult female White Leghorn chickens (*Gallus gallus*) were randomly divided into five groups: a vehicle control and four mesterolone treatment groups (4, 8, 12, and 16 mg/kg). Mesterolone was administered orally every three days for four weeks. Immunocytochemical techniques and morphometric analyses were employed to measure the changes in muscle weight, fiber size, satellite cell (SC) composition, and number of myonuclei. Mesterolone increased both body and muscle weights and induced hypertrophy in glycolytic PM fibers but not in oxidative ALD fibers. Mesterolone induced SC proliferation in both muscles; however, the myonuclear accretion was noticeable only in the PM muscle. In both muscles, the collective changes maintained a constant myonuclear domain size and the changes were dose independent. In conclusion, mesterolone induced distinct dose-independent effects in avian oxidative and glycolytic skeletal muscle fibers; these findings might be clinically valuable in the treatment of age-related sarcopenia.

## Introduction

Avian skeletal muscles are composed of basal lamina-enclosed muscle fibers that can be divided into three major types according to their ultrastructure, metabolic characteristics, and contractile dynamics: (i) type I red slow-twitch oxidative fibers, (ii) type IIa intermediate fast-twitch oxidative-glycolytic fibers, and (iii) type IIb white fast-twitch glycolytic fibers^1^. Skeletal muscles are either heterogeneous (composed of multiple muscle fiber types) or homogeneous (composed predominantly of the same fiber type)^2^. In chickens, the *anterior latissimus dorsi* (ALD) and *pectoralis major* (PM) are homogeneous muscles. The ALD muscle is a fatigue-resistant slow-contracting (tonic) muscle composed exclusively of oxidative type I fibers, whereas the PM muscle is an easily fatigable fast-contracting (phasic) muscle composed almost entirely of glycolytic type IIb fibers^3–5^. The oxidative and glycolytic muscle fibers exhibit distinct responses under different physiological and pathological conditions. These distinct responses are usually accompanied by unique changes in the morphological and cellular characteristics of the fiber^6–8^.

Satellite cells (SCs) are mononuclear adult myogenic stem cells located between the outer basal lamina and sarcolemma of muscle fibers^4,9^. They are responsible for postnatal muscle growth, repair, and regeneration^10^. When there is no need for growth or repair, SCs remain in a quiescent state. However, they become active in response to various stimuli (such as neural activity, exercise loading, and hormones), begin proliferating, and finally differentiate into post-mitotic myonuclei (MN). In fact, SCs contribute to postnatal muscle growth, repair, and regeneration via two mechanisms: (*i*) by fusing into preexisting mature muscle fibers (hypertrophy), a process known as myonuclear accretion, or less commonly (*ii*) by fusing together and forming nascent muscle fibers (hyperplasia)^9^. SCs play an indispensable role during early postnatal muscle fiber growth; however, they are dispensable for adult skeletal muscle fiber growth^11^.

Among the various stimuli promoting SC activity are anabolic androgenic steroids (AASs), which also augment muscle mass and size^4,5,12^. Mesterolone (17-beta-hydroxy-1 alpha-methyl-5 alpha-androstan-3-one) is one of the most frequently used exogenous AASs for the treatment of hypogonadism^12,13^. Mesterolone is distinguished from other AASs by its unique chemical structure that does not undergo ring aromatization into estrogen therefore it has no estrogenic side effects. Moreover, mesterolone has a very low hepatotoxicity, and confers oral activity that allows its oral route of administration^14^. These advantages of mesterolone encourage it to be considered as a potential alternative therapy for age-related sarcopenia instead of injectable AASs. A previous study reported that mesterolone significantly increases the weight and fiber size of the soleus muscle of transgenic mice^15^. We found that oral administration of mesterolone significantly increases the number of SCs in maturing avian pectoralis, a response accompanied by increased myonuclear density and DNA content^12^. However, it is unclear if mesterolone has distinct effects on different types of normal adult muscle fibers.

In this study, we evaluated the dose-related and fiber type-specific effects of mesterolone on two distinct adult avian skeletal muscles, the predominantly oxidative ALD and the mainly glycolytic PM. We hypothesized that mesterolone differentially influences these two muscles by inducing distinct changes in their fiber morphometric and cellular characteristics.

## Results

### Body and muscle weights

**Figure 1:**
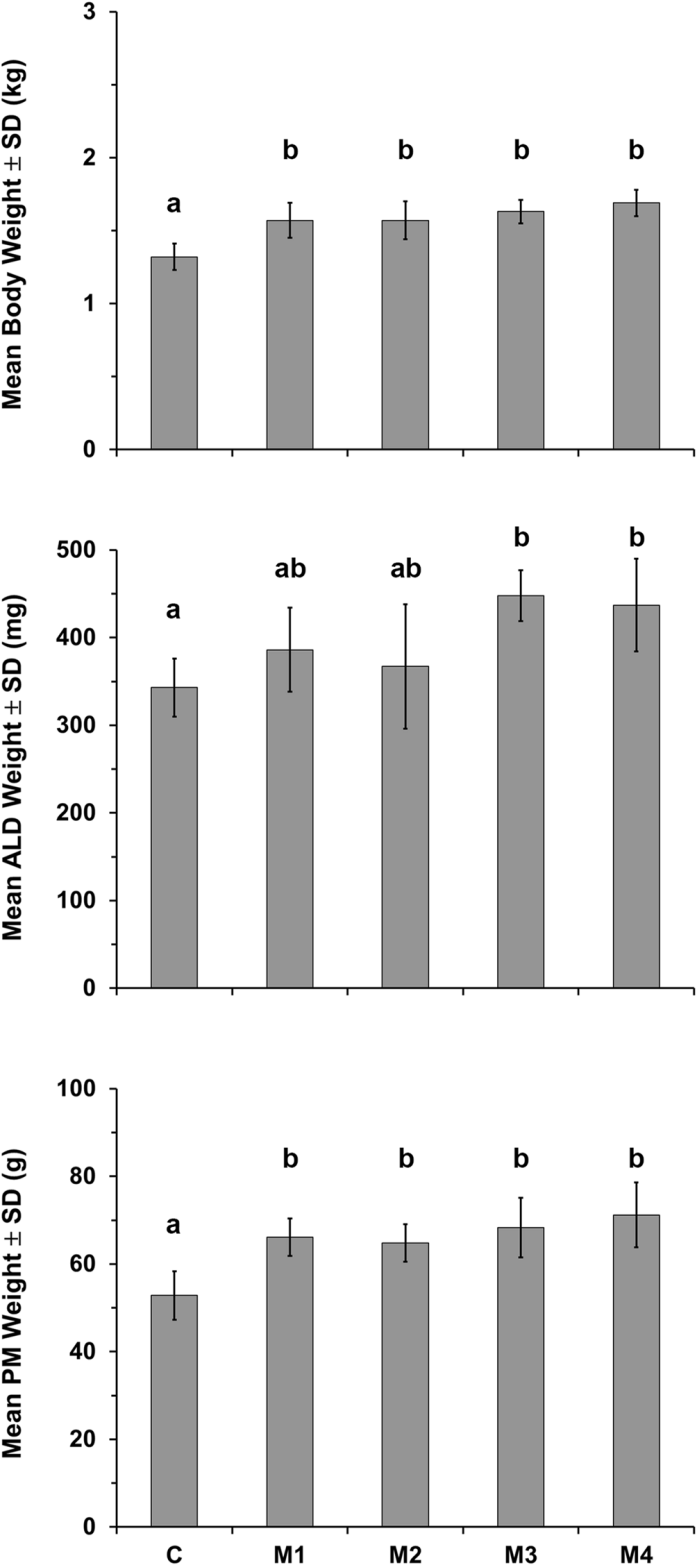
Body and muscle weights in control and mesterolone-treated groups. C: control group; M1, M2, M3, M4: groups treated with 4, 8, 12, and 16 mg/kg mesterolone, respectively. ALD: anterior latissimus dorsi muscle; PM: pectoralis major muscle; SD: standard deviation. Different alphabets (**a**, **b**) indicate significant difference among the groups (ANOVA, LSD *post-hoc*).

All four mesterolone dose groups demonstrated significantly (*P* < 0.01) greater mean body weight than the control group (control group: 1.32 ± 0.09 kg; M1: 1.57 ± 0.12 kg; M2: 1.58 ± 0.13 kg; M3: 1.63 ± 0.08 kg; M4: 1.69 ± 0.09 kg), but there were no significant differences among the mesterolone dose groups, which implies that the effect was dose independent (Fig. 1). Additionally, both ALD (*P* < 0.05) and PM (*P* < 0.01) muscle weights increased significantly following mesterolone administration (Fig. 1).

### Fiber size

There were no significant differences in the mean cross-sectional area (CSA) and fiber diameter between the ALD muscle fibers of control and mesterolone-treated groups or among the mesterolone-treated groups (Table 1). However, the mean CSA and fiber diameter of PM fibers were significantly (*P* < 0.05) greater in all mesterolone-treated groups than in the control group (Table 2). There were no significant differences in the CSA and diameter of PM fibers among the mesterolone-treated groups (Table 2). The comparative changes between ALD and PM in fiber size are summarized in Table 3. In summary, mesterolone induced hypertrophy in glycolytic PM fibers, but not in the oxidative ALD fibers; these hypertrophic effects of mesterolone on PM muscle fibers were dose independent.

**Table 1:** *Anterior latissimus dorsi* fiber size and cellular parameters in control and mesterolone-treated groups.

Parameter	Control	M1	M2	M3	M4
Fiber CSA (µm^2^)	1817 ± 319	2047 ± 323	1836 ± 156	2127 ± 245	2038 ± 526
Lesser fiber diameter (µm)	37.32 ± 4.11	39.82 ± 3.16	37.94 ± 1.83	40.17 ± 2.32	39.26 ± 5.44
SCs per 100 fibers	22.8 ± 3.6	42.5 ± 7.0**	35.3 ± 6.5*	44.3 ± 7.6**	35.5 ± 4.9**
SCs per 1 mm	10.8 ± 1.7	21.0 ± 3.5**	17.9 ± 3.3**	21.1 ± 3.6**	17.7 ± 2.5**
SC frequency (%)	5.9 ± 0.5	9.4 ± 2.4**	9.1 ± 1.7*	8.6 ± 1.6*	8.8 ± 1.6*
Surface area of plasma- lemma per SC (µm^2^)	10,938 ± 632	6044 ± 912**	6851 ± 1331**	6054 ± 745**	7031 ± 1068**
MN per 100 fibers	363.3 ± 65.0	418.8 ± 78.9	353.8 ± 20.8	477.0 ± 75.0	369.5 ± 42.0
MN per 1 mm	171.5 ± 30.5	208.7 ± 39.3	170 ± 10	223.3 ± 35.2	176.2 ± 20
MND (µm^3^)	10,634 ± 1234	9926 ± 1591	10,830 ± 1135	9619 ± 1042	11,454 ± 1969

M1, M2, M3, M4: groups treated with 4, 8, 12, and 16 mg/kg mesterolone, respectively. CSA: cross-sectional area; SC: satellite cell; MN: myonuclei; MND: myonuclear domain. Values represent the means ± standard deviations. *P < 0.05, **P < 0.01 significantly different from the control group (ANOVA, LSD *post-hoc*).

**Table 2:** *Pectoralis major* fiber size and cellular parameters in control and mesterolone-treated groups.

Parameter	Control	M1	M2	M3	M4
Fiber CSA (µm^2^)	2042 ± 244	2616 ± 267**	2718 ± 186**	2390 ± 22*	2631 ± 133**
Lesser fiber diameter (µm)	42.2 ± 2.4	48.3 ± 2.4**	48.4 ± 2.1**	46.9 ± 1.2*	46.8 ± 1.1*
SCs per 100 fibers	8.0 ± 1.0	20.3 ± 4.8**	16.5 ± 3.4*	20.7 ± 4.6**	15.5 ± 4.5*
SCs per 1 mm	3.9 ± 0.4	10.0 ± 2.4**	8.1 ± 1.7*	10.2 ± 2.3**	7.6 ± 2.2*
SC frequency (%)	3.3 ± 0.3	6.3 ± 1.3**	5.0 ± 0.7*	6.0 ± 1.0**	4.8 ± 1.3*
Surface area of plasmalemma per SC (µm^2^)	144,936 ± 22,869	67,980 ± 18,521**	81,927 ± 14,250**	63,640 ± 11,869**	86,245 ± 19,336**
MN per 100 fibers	235.8 ± 14.3	300.5 ± 25.4**	313 ± 22.1**	321.3 ± 22.9**	309.8 ± 10.7**
MN per 1 mm	114.5 ± 6.9	146.0 ± 12.3**	152.1 ± 10.8**	156.1 ± 11.1**	150.6 ± 5.2**
MND (µm^3^)	17,930 ± 2951	17,998 ± 2170	17,904 ± 1244	15,382 ± 1077	17,488 ± 1076

M1, M2, M3, M4: groups treated with 4, 8, 12, and 16 mg/kg mesterolone, respectively. CSA: cross-sectional area; SC: satellite cell; MN: myonuclei; MND: myonuclear domain. Values represent the means ± standard deviations. *P < 0.05, **P < 0.01 significantly different from the control group (ANOVA, LSD *post-hoc*).

**Table 3:** Distinct mesterolone-induced changes in fiber size and cellular parameters of *anterior latissimus dorsi* (ALD) and *pectoralis major* (PM) muscles.

Parameter	ALD muscle (oxidative fibers)	PM muscle (glycolytic fibers)
Fiber CSA	Unchanged	> 17–33%
Lesser fiber diameter	Unchanged	> 11–15%
SCs per 100 fibers	> 1.5–1.9 fold	> 1.9–2.6 fold
SCs per 1 mm	> 1.6–2.0 fold	> 1.9–2.6 fold
SC frequency	> 1.5–1.6 fold	> 1.5–1.9 fold
Surface area of plasmalemma per SC	< 1.6–1.8 fold	< 1.7–2.3 fold
MN per 100 muscle fibers	Unchanged	> 1.3–1.4 fold
MN per 1 mm	Unchanged	> 1.3–1.4 fold
Myonuclear domain	Unchanged	Unchanged

CSA: cross-sectional area; SC: satellite cell; MN: myonuclei.

### Cellular parameters (SCs and MN)

**Figure 2:**
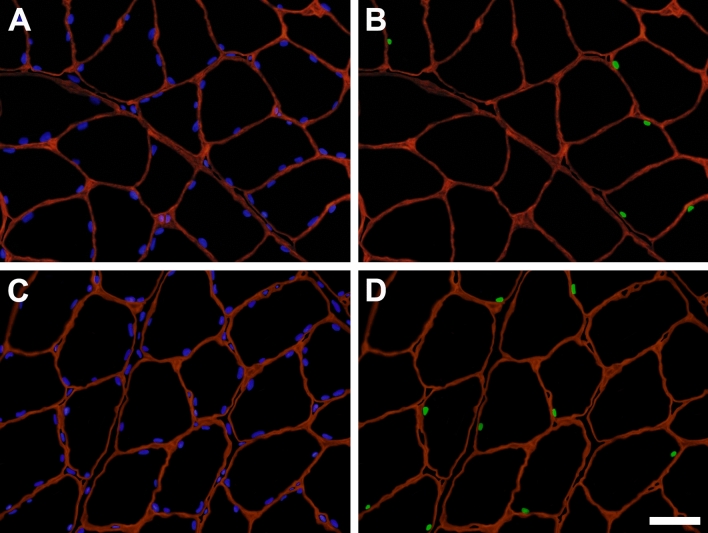
Immunofluorescent identification of satellite cell nuclei (SCN) in cross sections of anterior latissimus dorsi muscles obtained from control and mesterolone-treated chickens. (**A**) and (**B**) represent a section obtained from a control bird. (**C**) and (**D**) represent a section obtained from a mesterolone-treated bird. (**A**) and (**C**) show all nuclei in blue (DAPI staining) and the basal laminae of muscle fibers in red (anti-laminin staining). (**B**) and (**D**) show SCN in green (anti-Pax7 labeling) and the basal laminae in red. Scale bar = 30 μm.

**Figure 3:**
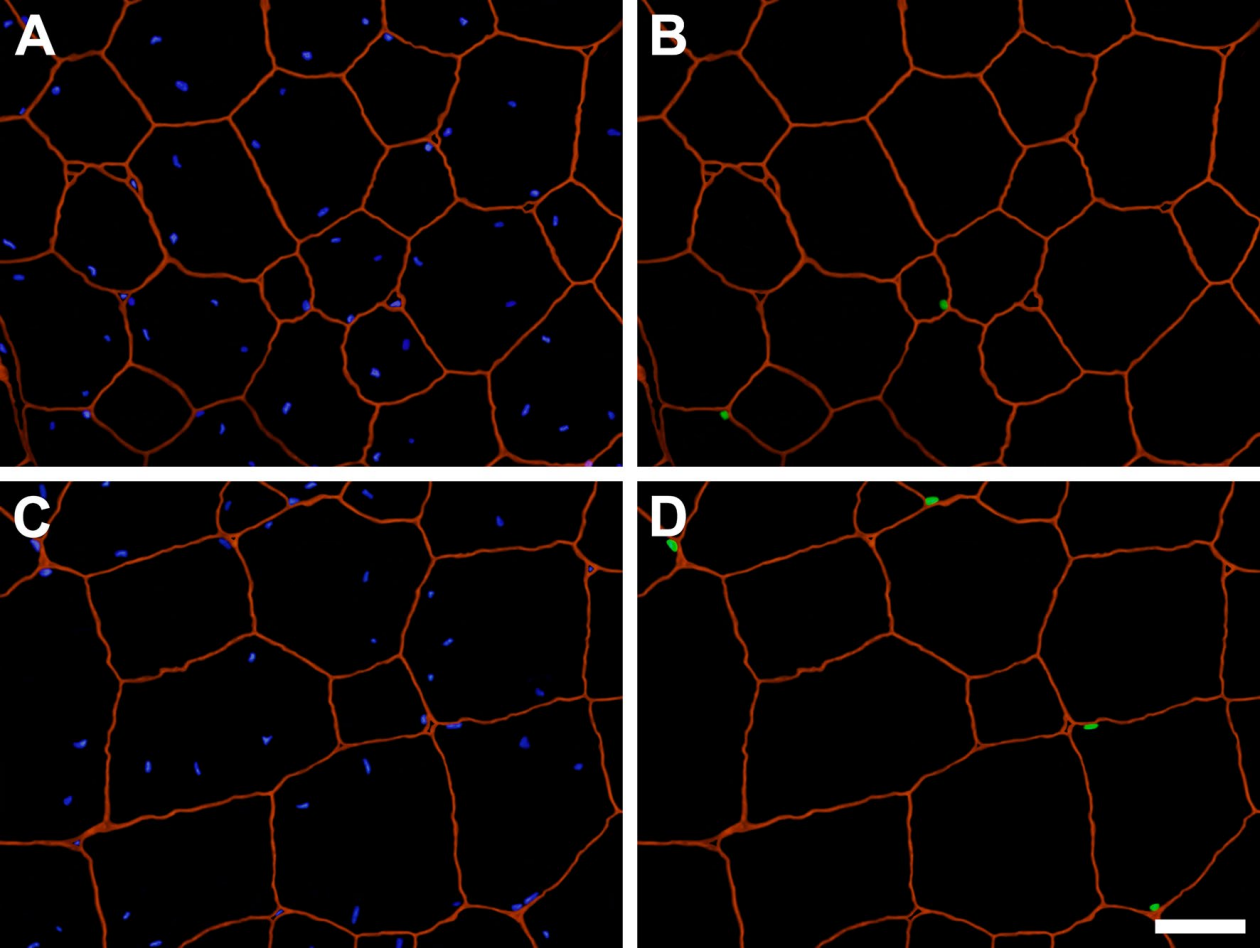
Immunofluorescent identification of satellite cell nuclei (SCN) in cross sections of pectoralis major muscles obtained from control and mesterolone-treated chickens. (**A**) and (**B**) represent a section obtained from a control bird. (**C**) and (**D**) represent a section obtained from a mesterolone-treated bird. (**A**) and (**C**) show all nuclei in blue (DAPI staining) and the basal laminae of muscle fibers in red (anti-laminin staining). (**B**) and (**D**) show SCN in green (anti-Pax7 labeling) and the basal laminae in red. As a unique histological feature of the chicken pectoralis, myonuclei are normally found deep within the fiber sarcoplasm^12^. Scale bar = 30 μm.

SC indices (SCs per 100 fibers, SCs per unit fiber length, SC frequency, and SC concentration) and myonuclear indices (MN per 100 fibers, and MN per unit fiber length) were measured to assess the effects of mesterolone on the cellular parameters of the fibers. In the basal state (control group), ALD muscle fibers contained more SCs and MN than PM muscle fibers (Tables 1 and 2). Mesterolone significantly (*P* < 0.05) increased all SC indices in both ALD and PM muscles, when compared to those in the vehicle-treated muscles (Tables 1 and 2). However, there were no significant (*P* > 0.05) differences in SC indices among the mesterolone-treated groups for each muscle. This indicated that mesterolone induced SC proliferation in both PM and ALD muscle fibers in a dose-independent manner. Immunofluorescence sections labeled for SC nuclei from ALD and PM muscles are shown in Fig. 2 and Fig. 3, respectively. The SC indices increased to a relatively greater extent in PM fibers than ALD fibers (Table 3).

On the other hand, Mesterolone significantly (*P* < 0.01) increased myonuclear indices in the PM muscle (Table 2) but did not induce any significant (*P* > 0.05) changes in those of the ALD muscle (Table 1). There were no significant (*P* > 0.05) differences in the myonuclear indices of the PM muscle among the mesterolone-treated groups. Collectively, these findings suggest that the increase in SC number within PM muscle fibers resulted in a higher rate of SC-mediated myonuclear accretion, while the increase of SCs in the ALD muscle fibers failed to augment myonuclear accretion. Additionally, mesterolone induced myonuclear accretion in the PM muscle in a dose-independent manner.

### Myonuclear domain

The size of the myonuclear domain (MND) was calculated in both ALD and PM muscles. There were no significant (*P* > 0.05) changes in the MND size in both ALD and PM muscles in response to mesterolone administration (Tables 1, 2, and 3). However, the MND size in these two muscle types was maintained by two different mechanisms. In PM, the increase in the fiber size (hypertrophy) was compensated by addition of new myonuclei (myonuclear accretion) to maintain the size of the MND. Alternatively, in ALD, the MND remained constant because there was no fiber hypertrophy and no myonuclear accretion.

## Discussion

This study demonstrates the differential effects of the AAS mesterolone on oxidative ALD muscle fibers and glycolytic PM muscle fibers in adult chicken. Mesterolone induced fiber hypertrophy in the PM muscle but not in the ALD muscle. Additionally, it triggered significant increases in SC numbers in both PM and ALD muscle. However, this resulted in an increased muscle fiber myonuclear content only in the PM. The increased myonuclear accretion, coupled with the hypertrophic changes, maintained a constant MND in the PM muscle following mesterolone treatment. However, the SC increase in the ALD muscle failed to induce myonuclear accretion; there was no significant change in muscle fiber size in the ALD and, therefore, MND remained unaltered.

Adult oxidative and glycolytic muscles normally contain different proportions of SCs and myonuclei, both of which are more abundant and concentrated within oxidative fibers at rest than in the glycolytic fibers^16,17^. We confirmed this in the current study by using robust immunocytochemical techniques and precise morphometric and cellular analyses.

The response of skeletal muscles to AASs varies according to fiber type, androgen receptor density, AAS type, AAS dosage, mode and duration of administration, sex, age, and muscle activity level^18–20^. In this study, we examined only female chickens to avoid possible confounding effects of the relatively higher and dynamic levels of endogenous androgens in males^21^. In a previous study, we showed that mesterolone induced significant growth in maturing PM muscle and that this response was associated with increase in SC numbers^12^. Here, we demonstrate comparable results in the adult PM. In a previous study, we also reported that the injectable AAS, sustanon, significantly boosted SC number and increased the overall size of the ALD muscle fibers in adult female chicken^5^. This was in line with the previous findings that injectable AASs are more potent than oral AASs, such as mesterolone^22^. However, it cannot be ignored that the injectable steroids are associated with significant side effects. For example, Sustanon has been associated with estrogenic, hepatic, and cardiovascular side effects, whereas mesterolone has not been related to any of these toxic effects^14^. Mesterolone has only been found to induce dyslipidemia when used at a very high dose (100 mg/d) for a long period of time (6 months)^23^.

Skeletal muscles grow through either only hypertrophy or both hypertrophy and hyperplasia^24^. In this study, we could not exclude the possibility of mesterolone-induced hyperplasia of the ALD muscle. However, the contribution of hyperplasia to muscle growth is minimal, when compared to that of hypertrophy, in vertebrate skeletal muscle^25,26^. Splitting of preexisting muscle fibers and connective tissue invasion was proposed as an alternative mechanism for muscle growth. However, this is usually observed only in muscles under stretch overload or in actively regenerating muscles^27,28^. We did not observe fiber splitting in any of the large number of images acquired for this study.

The myonuclear domain remains constant during fiber and muscle growth as MN expansion is offset by the increase in cytoplasmic volume, while the myonuclear domain is reduced by muscle atrophy^29^. However, this ratio is maintained only under certain conditions and certain studies have found a loose association between cytoplasmic volume and myonuclear number^30,31^. This discrepancy may arise from differences in the experimental model and the type of stimulus applied to induce muscle growth^32,33^. We found that the MND remained unaltered with mesterolone treatment in both ALD and PM muscles, albeit through different mechanisms.

Age-related sarcopenia (ARS) is a common condition, where there is a loss of skeletal muscle bulk because of aging, resulting in progressive impairment of physical activity. ARS can appear in individuals as early as their 30s and worsen progressively, causing an average loss of 40% of body muscle mass by the age of 80^34^. Glycolytic muscles are more susceptible to ARS^35^. The current management approaches for ARS in the elderly are limited^36^. Injectable testosterone replacement therapy is beneficial to restore muscle mass in individuals with ARS. However, the efficacy of injectable testosterone is limited by its serious side effects, such as increased risks of hepatotoxicity and cardiovascular diseases^37^. In addition, we reported that the effect of injectable testosterone complex, Sustanon, on skeletal muscles is type non-specific (results in glycolytic and oxidative muscle fiber hypertrophy)^5,38^. However, the beneficial effect of mesterolone on skeletal muscle is fiber type-specific (only glycolytic fibers). The effect is dose independent, implying that a minimum dose can be an effective therapy for ARS with minimal side effects when compared to injectable testosterone.

Comparative studies at the subcellular and molecular levels are needed to better understand the mechanisms underlying these differential responses of oxidative and glycolytic skeletal muscle fibers to mesterolone. Also, additional studies for investigating the possibility of hyperplasia in ALD following mesterolone treatment, such as immunostaining experiments with antibodies against proteins expressed specifically in newly formed fibers, are needed.

In conclusion, this study showed that mesterolone induced a dose-independent hypertrophy in the glycolytic fibers but not in the oxidative fibers. In addition, mesterolone induced dose-independent SC-mediated myonuclear accretion only in the glycolytic fibers. This study provides further evidence that oxidative and glycolytic muscle fibers exhibit distinct differential responses under AASs’ stimulation. Importantly, this study suggests that a minimum dose of mesterolone might be clinically effective in treating ARS, since ARS mostly affects glycolytic fibers^39^.

## Methods

### Experimental model

Adult female White Leghorn chickens (*Gallus gallus*) were raised in the animal care unit at Jordan University of Science and Technology (JUST) in accordance with the institutional guidelines. The birds were housed in large cages (4 birds per cage) at a controlled room temperature of 21 °C, fed ad libitum and under a 12–12 h light–dark cycle. All animal care procedures and treatments were conducted with the approval of the JUST committee on animal care, and in accordance with the guidelines of the U.S. National Institutes of Health on the use and care of laboratory animals and with the Animal Research: Reporting of In Vivo Experiments (ARRIVE) guidelines (https://arriveguidelines.org).

At the start of 26 weeks of age, the birds were randomly divided into 5 groups (*n* = 4 in each group): a control (C) group receiving distilled water (vehicle) only and four mesterolone dose groups receiving 4, 8, 12, and 16 mg/kg (termed groups M1 to M4, respectively). Mesterolone treatments were provided once every three days for four weeks based on a previous protocol^12^. Each dose was dissolved in 1 mL distilled water before being administered by oral gavage using a feeding needle. Animals were weighed after group allocation (baseline), at the end of each week, and finally before sacrifice.

At the start of the 30th week of age, the birds were sacrificed by cervical dislocation, and the left ALD and PM muscles were dissected out, trimmed free of fat, and weighed. The chicken ALD is a small superficial muscle located on the dorsal surface of the thorax originating from a number of cervical and thoracic vertebrae and inserting into the humerus, where it acts to maintain the folded wing against the body. Therefore, it is a fatigue-resistant muscle composed entirely of oxidative type I fibers^3,5^. The PM is a relatively large muscle that extends from the sternum to the humerus, where it provides the abrupt force required for the wing down-stroke during flapping flight. It is an easily fatigable fast (phasic) contracting muscle that is composed almost entirely (> 99%) of glycolytic type IIb (white) fibers^4,40^.

### Tissue preparation and sectioning

Muscle samples, approximately 0.5 × 0.5 × 1 cm, were cut from each muscle, embedded in optimal cutting temperature compound (O.C.T.; Bio-Optica, Milano, Italy), frozen in 2-methylbutane, cooled using liquid nitrogen, and stored at − 80 °C. Serial cross-sections of 10-µm thickness were cut at − 20 °C using a cryostat (SLEE-MNT, SLEE Medical GmbH, Mainz, Germany). Each pair of serial sections was picked up on positively charged microscopic slides (AM-1800, AmLabs West LLC, Boulder, CO, USA). Two sections were collected on each slide to increase the possibility of obtaining better fields for imaging. Serial slides bearing sections were numbered and stored at − 40 °C. Longitudinal sections were also obtained to measure the lengths of SC nuclei and MN.

### Immunohistochemistry

Slides were recovered from − 40 °C storage, air-dried for 10 min, fixed with 4% formaldehyde in 1× phosphate-buffered saline (PBS) solution for 4 min, washed twice in fresh PBS (5 min/wash), and incubated in ice-cold methanol for 15 min for permeabilization, followed by two 5 min washes with PBS. Blocking solution, consisting of 5 mM ethylenediaminetetraacetic acid (EDTA), 1% bovine serum albumin (BSA) diluted in PBS, and 5% goat serum, was applied over the sections for 20 min. Slides were then drained of the blocking solution and incubated with 150 μL fresh blocking solution containing a mouse monoclonal antibody against chicken Pax7 (1:100, Developmental Studies Hybridoma Bank, Iowa City, Iowa, USA) to label SC nuclei, and a rabbit polyclonal antibody against mouse laminin (1:200, L9393, Sigma Chemical Co., St. Louis, MO) to label the muscle fiber basal lamina. Slides were incubated overnight at 4 °C in this primary antibody solution.

Following day, the slides were washed twice in 1× PBS at room temperature (5 min/wash), and incubated for 45 min in PBS containing Alexa Fluor 488-conjugated goat anti-mouse secondary antibody (1:400, A-11001, Invitrogen, Thermo Fisher Scientific, Carlsbad, CA, USA) to label anti-Pax7 and a tetramethylrhodamine (TRITC) goat anti-rabbit secondary antibody (1:400, T6778, Sigma-Aldrich, Merck, Germany) to label anti-laminin. Slides were then washed twice in 1× PBS for 5 min each, and mounted with UltraCruz Mounting medium containing DAPI (SC-24941, Santa Cruz Biotechnology Inc., Dallas, TX, USA) for nuclear counterstaining and sealed under cover slips.

### Imaging

Four different fields of view from each slide were captured at 200 × using an AxioScope.A1 microscope equipped with epifluorescence and an Axiocam ICc5 digital still camera (Carl-Zeiss, Jena, Germany). The captured fields were numbered in order of acquisition for each animal. Three epifluorescence images per field showing DAPI nuclear staining in blue, Alex Fluor-488 staining of SCs in green, and TRITC staining of the basal lamina in red were acquired for each field, and superimposed using Adobe Photoshop (Adobe System Inc., San Jose, CA, USA).

### Image analyses

Fiber morphometric measurements were assessed using the ImageJ software (version 1.52a, available at: http://rsbweb.nih.gov/ij/). The actual cross-sectional area and the ellipse minor axis, which is equal to the lesser fiber diameter, were measured for 300 contiguous fibers.

The numbers of SC nuclei and MN were counted for 300 contiguous fibers. SC nuclei were identified as all Pax7^+^/DAPI^+^ nuclei within the basal lamina, while MN were all Pax7^-^/DAPI^+^ nuclei inside the basal lamina. The numbers of SC nuclei and MN per unit length (mm) of muscle fiber were calculated using the formula: N = A/(Ln + M), where A is the mean number of nuclei per fiber cross section, Ln is the mean length of the nucleus and M is the thickness of the tissue section on the slide^4,5,10^. Longitudinal sections were used to measure the lengths of both SC nuclei and MN. Similar immunofluorescence techniques were employed to study these longitudinal sections. The mean lengths of 50 SC nuclei and 100 MN from each bird were measured using ImageJ.

The frequency of SCs (the percentage of SC nuclei to all nuclei located under the basal lamina) was calculated using the formula: SC nuclei/(SC nuclei + MN) × 100%. The concentration of SCs (a measure of aggregation) is measured as the surface area of plasmalemma per SC. SCs become more concentrated and closer to each other as the surface area of plasmalemma per SC decreases. The surface area of plasmalemma per SC (S) can be calculated as S = πEU/N, where E is the lesser diameter (ellipse minor axis), U is the unit length of fiber (1 mm), and N is the number of SCs per unit length of muscle fiber^4,5,10^. Finally, the myonuclear domain (MND), which is the volume of cytoplasm per myonucleus, was calculated by dividing the volume of sarcoplasm per unit length of fiber by the number of myonuclei in this unit length^5^.

### Statistical analysis

All numerical data were expressed as mean ± standard deviation (SD). Samples were divided into five groups (a control and four mesterolone-treated groups). Levene’s test was first applied to determine the homogeneity of variance among the groups. Data were then evaluated using one-way analysis of variance (ANOVA) at 5% level of significance. If a significant difference was found, Fisher’s least significant difference (LSD) *post-hoc* test was used to examine the exact statistical difference between the groups.

### Ethics declaration

This study was conducted with the approval of the Animal Care and Use Committee at Jordan University of Science and Technology, and in accordance with the guidelines of the U.S. National Institutes of Health on the use and care of laboratory animals and with the Animal Research: Reporting of In Vivo Experiments (ARRIVE) guidelines (https://arriveguidelines.org).

## Data Availability

The datasets generated during and/or analysed during the current study are available from the corresponding author on reasonable request.
